# The value of serum pepsinogen levels for the diagnosis of gastric diseases in Chinese Han people in midsouth China

**DOI:** 10.1186/1471-230X-14-3

**Published:** 2014-01-03

**Authors:** Xiao-mei Zhang, Jia-xin Li, Gui-ying Zhang, Xin-hua Li, Huan Gu

**Affiliations:** 1 Department of Gastroenterology, Xiangya Hospital, Central South University, Changsha 410008, Hunan Province China; 2 Department of Pediatrics, Huai'an First People’s Hospital, Nanjing Medical University, 6 Beijing Road West, Huai'an 223300, China

**Keywords:** Gastritis, Stomach, Gastric cancer, Peptic ulcer, Serum pepsinogen

## Abstract

**Background:**

Serum pepsinogen (PG) levels are valuable in the diagnosis of gastric diseases. However, PG levels are affected by many factors such as the area and race. This study aimed to investigate serum PG levels in patients with different gastric diseases who were Chinese Han people in Hunan Province, midsouth China.

**Methods:**

A total of 248 gastric disease patients and 34 healthy controls were enrolled. The patients included those with non-atrophic and chronic atrophic gastritis, gastric and duodenal ulcer, early and advanced gastric cancer. Serum PG I and II levels were detected by Biohit ELISA kit (Finland), and PG I/II ratio was calculated. Differences in patients with gastric disease and healthy controls were analyzed using paired t-test.

**Results:**

Compared with controls, patients with early and advanced gastric cancer had a significantly lower PG I level and PG I/II ratio (p <0.005). In contrast, patients with gastric and duodenal ulcer had a significantly higher PG I level (p <0.005). Compared with atrophic gastritis patients, patients with early and advanced carcinoma of the stomach had a significantly lower PG I/II ratio (p < 0.001). Combination of the cut-off levels of PG I (70 μg/L) and PG I/II ratio (6) provided 62.1% sensitivity of and 94.2% specificity for the diagnosis of gastric cancer.

**Conclusions:**

Decreased PG I level and PG I/II ratio are risk factors for gastric cancer. Combined use of serum PG I level and PG I/II ratio may help the early diagnosis of gastric cancer.

## Background

Pepsinogens (PG) are aspartic proteinases which are mainly secreted by gastric cells. PG can be classified into two biochemically and immunologically distinct types: pepsinogen I (PGI) and pepsinogen II (PGII). PG I is secreted only from the gastric fundic mucosa, while PGII is secreted from the cardiac, fundic, and antral mucosa of the stomach, and also from the duodenal mucosa [1,2]. PGs are also released into the circulation and it is widely accepted that serum PG level reflects the functional and morphologic status of stomach mucosa. Human pepsinogens have a diagnostic value for various gastroduodenal disorders, especially for peptic ulcer, atrophic gastritis and gastric cancer [3-8]. The pepsinogen I/II ratio can provide even better information on the extent of chronic gastritis [4].

Gastric cancer is the second leading cause of cancer-related death in the world. However, the risk of gastric cancer varies among the countries and populations in the world. High risk areas include Korea, Japan and China [9]. When diagnosed at an early stage, 5-year survival rate for gastric cancer exceeds 90%, but the 5-year survival rates are below 50% when diagnosed at an advanced stage [10]. Thus, it is necessary to diagnose gastric cancer at an early stage to reduce the morbidity and mortality from gastric cancer.

Atrophic gastritis is a well-recognized high-risk condition for developing gastric cancer. The “gold standard” for the diagnosis of gastric atrophy is the histological study of biopsies obtained during an upper gastrointestinal endoscopy, an invasive method hardly suitable for population screening [11]. Recent reports showed that human serum PG levels were valuable in the diagnosis of gastric diseases, including gastric atrophy and gastric cancer [3-8]. In addition, the test for serum PG as a marker for chronic atrophic gastritis has been incorporated into gastric cancer screening programs, on a trial basis, to identify people who would benefit from gastric cancer screening [12,13].

However, PG levels are affected by many factors such as the area, race, age, gender, height, body weight, body surface area, smoking, drinking habits and *Helicobacter pylori* infection [3,4,7]. Unfortunately, few studies have examined serum PG changes in patients with different gastric diseases in midsouth China. Therefore, to provide a valuation of serum PG levels for the survey of gastric cancer in this area, in the present study we measured serum PG I and PG II levels in 34 healthy controls and 248 patients with various gastric diseases from Hunan Province, midsouth China.

## Methods

### Study population

The subjects were enrolled at the Department of Gastroenterology, Xiangya Hospital, Central South University from September 2005 to August 2007. They included 248 patients (163 men) with a mean age of 52.3 ± 12.3 years (range 19-80 years) who had upper abdominal complaints and evidence of gastroduodenal disorder, and 34 healthy controls (19 men) with a mean age of 52.4 ± 15.1 years (range 29-77 years) who had no upper abdominal complaints or evidence of gastroduodenal disorder and liver diseases. All of them were Chinese Han people in Hunan Province.

The study protocol was approved by the Ethics Committee of Central South University, and written informed consent was obtained from all participants.

### Determination of serum PG levels

Approximately 5 mL fasting blood was collected from each participant. The serum was separated and stored at -20°C until analyzed. PG I and II concentrations were detected by enzyme-linked immunosorbent assay kit (Biohit ELISA kit, Finland). The absorbance was measured by using a microplate spectrophotometer at 450 nm. PG I and II levels were calculated based on standard curve. The ratio of PG I/II was then calculated. Each sample was evaluated twice for each patient, a coefficient of variation of < 15% was considered acceptable.

### Endoscopic and clinicopathological examinations

All subjects underwent a gastroscopy and biopsy. The biopsies were scored semi-quantitatively by two histopathologists, according to the updated Sydney classification system. Gastrointestinal endoscopy was performed for the entire stomach. *H. pylori* infection was detected by rapid urease test and anti-*H. pylori* IgG test as described previously [11], and current *H. pylori* infection was confirmed if both rapid urease test and anti-*H. pylori* IgG test gave positive results. Experienced endoscopists performed each examination without knowledge about the serological data on the study subjects. Based on endoscopic examination and histological appearances, the patients were classified into seven categories as follows: 55 cases of non-atrophic gastritis (NAG), 20 cases of chronic atrophic gastritis (CAG), 36 cases of gastric ulcer (GU), 31 cases of duodenal ulcer (DU), 69 cases of advanced gastric cancer (AGC), 13 cases of early gastric cancer (EGC) before operation and 24 cases with partial gastrectomy for gastric cancer, one of them with recurrence of gastric cancer.

### Statistical analysis

Data were expressed as mean ± SD. Differences in patients with gastric disease and healthy controls were analyzed using Student’s t-test. The distribution of variables was tested by “Kolmogorov-Smirnov”. For normal distribution, the mean and standard of deviation values were analyzed for significant difference between the two groups using Student’s t-test. Otherwise, the median values were analyzed for significant difference using Mann-Whitney test. The statistical analysis was performed by using SPSS program for Windows version 17.0. p < 0.05 was considered statistically significant.

## Results

### Serum PG levels and PGI/II ratio in patients with different gastric diseases

By ELISA assay we detected serum levels of PG I and PG II and calculated PG I/II ratio for all subjects (Table 1). Compared with the normal control group, PG I and PG II levels and PG I/II ratio showed no statistical significance in NAG and CAG group (p > 0.05). However, PG I and PG II levels and PG I/II ratio were significantly higher in patients with duodenal ulcer than in the controls (p < 0.05). In addition, serum PGI level and PGI/II ratio were significantly lower in patients with early or advanced gastric cancer than in the controls and NAG group (p < 0.001), but there were no significant differences in PG I and PG II levels and PG I/II ratio between EGC and AGC group. Compared with CAG patients, serum PGI level was lower in patients with AGC and PG I/II ratio was lower in patients with AGC or EGC (p < 0.05). Notably, PG I and PG II levels were extremely low in the patients with partial gastrectomy for gastric cancer (p < 0.005), but were abnormally high in one patient with recurrence of gastric cancer after gastrectomy (Table 1).

**Table 1:** Serum PG I and II levels in various gastric disorders (n = 282)

**Group**	**N**	**PG I (μg/L)**	**PG II (μg/L)**	**PG I/PG II**
Healthy controls	34	118.39 ± 47.80	12.39 ± 5.90	11.74 ± 6.23
Non-atrophic gastritis	55	112.46 ± 51.71	12.57 ± 5.98	10.63 ± 5.74
Atrophic gastritis	20	93.63 ± 49.34	10.85 ± 4.58	11.07 ± 5.78
Early gastric cancer	13	71.48 ± 28.78^‡▼^	14.22 ± 4.90	5.19 ± 1.70^‡†^
Advanced gastric cancer	69	53.39 ± 34.03^‡†^	12.29 ± 5.63	4.88 ± 3.76^‡†^
Gastric ulcer	36	147.58 ± 57.81^▲†^	15.60 ± 13.42	14.47 ± 13.02
Duodenal ulcer	31	217.43 ± 51.12^‡†^	21.90 ± 19.45^▲†^	18.57 ± 16.63^▲†^
Gastrectomy	23	40.70 ± 15.38^‡*^	8.52 ± 4.52	4.43 ± 2.38^‡^
Recurrence after gastrectomy	1	289.32	65.89	4.39

Data were shown as mean ± SD.

^‡^p < 0.005, ^▲^p < 0.05 vs. Healthy controls; ^†^p < 0.005, ^▼^p < 0.05 vs. NAG; ^*^p < 0.05 vs. CAG.

### Low PG I level and PG I/II ratio could predict gastric cancer

**Figure 1:**
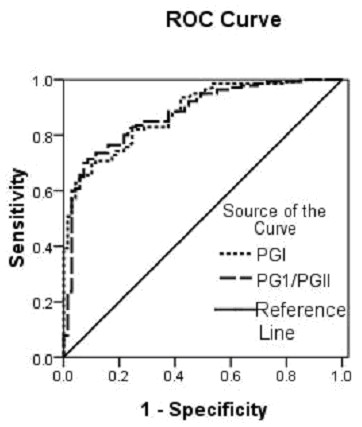
**Characteristic curves of PG I level and PG I/II ratio for discriminating gastric cancer.** The optimal cut-off points of PG I level and PG I/II ratio were determined by analysis of receiver operating characteristic (ROC). The discriminatory ability did not differ between PG I and PG I/II values, with areas under the ROC curves of 0.880 and 0.876, respectively (p = 0.28).

Next we investigated the optimal cut-off points and performance of PG I level and PG I/II ratio for the diagnosis of GC (Tables 2 and 3, Figure 1). We found that discriminatory ability did not differ between PG I and PG I/II values, with areas under the ROC curves of 0.880 and 0.876, respectively (p = 0.28). The optimal PG I cut-off concentration was 70.1 μg/L, with a sensitivity of 82.1% and a specificity of 72.5%. The optimal cut-off PG I/II ratio was 6.0, with a sensitivity of 82.9% and a specificity of 76.8%. PG I level had positive predictive value of 68.2%, a negative predictive value of 82.3%, and an accuracy of 77.2%.

**Table 2:** Comparison of frequency of PG levels in non-GC and GC groups

**Variables**	**Non-GC group (%)**	**GC group (%)**
**Number of subjects**	142	82
PG I (μg/L)		
≤30	2 (1.4)	27 (32.9)
≤40	2 (1.4)	33 (40.2)
≤50	9 (6.3)	43 (52.4)
≤60	19 (13.4)	47 (57.3)
≤70	25 (17.6)	56 (68.2)
≤80	33 (23.2)	61 (74.3)
≤90	41 (28.9)	67 (81.7)
>90	101 (71.1)	15 (18.3)
PG I/II radio		
≤3	2 (1.4)	21 (25.6)
≤4	7 (5.0)	31 (37.8)
≤5	18 (12.7)	43 (52.4)
≤6	25 (17.6)	53 (64.6)
≤7	33 (23.2)	56 (68.3)
≤8	38 (26.8)	61 (74.4)
≤9	49 (34.5)	65 (80.4)
>9	91(64.1)	4 (4.7)

Non-GC group included 55 of non-atrophic gastritis, 20 of atrophic gastritis, 36 of gastric ulcer, 31 of duodenal ulcer. GC group included 13 of early gastric cancer, 69 of advanced gastric cancer.

**Table 3:** Predicting gastric cancer based on serum PG I level and PG I/II ratio

	**PG I level**	**PG I/II ratio**
Area under the ROC curve	0.880 (0.835-0.925)	0.876 (0.827-0.925)
Optimal cult-off value	70.1 μg/L	6
Sensitivity, %	82.1	82.9
Specificity, %	72.5	76.8
Positive predictive value, %	68.2	64.6
Negative predictive value, %	82.3	82.3
Accuracy, %	77.2	75.8

Using a level of 70 μg/L PG I as a serologic cut-off for gastric cancer, we found that 68.2% patients with PG I < 70 μg/L and 31.8% patients with PG I ≥ 70 μg/L had gastric cancer. Using a cut-off of 6 for PG I/II ratio, we found that 76.8% patients with PG I/II < 6 and 23.2% patients with PG I/II ≥ 6 had tumors. Combining the two indexes of serum PG I ≤ 70 ug/L and PG I/PGII ≤ 6, we found that the diagnostic sensitivity and specificity of gastric cancer were 62.1% and 94.2%, respectively (Table 4). These results suggest that low PG I level and PG I/II ratio are valuable serologic markers for predicting gastric cancer when used in combination.

**Table 4:** Comparison of the diagnostic performance for gastric cancer based on different values of PG I level and PG I/II ratio

	**Sensitivity (%)**	**Specificity (%)**
PGI ≤ 70 and PGI/PGII ≤ 4	40.2	98.5
PGI ≤ 70 and PGI/PGII ≤ 5	54.8	96.4
PGI ≤ 70 and PGI/PGII ≤ 6	62.1	94.2
PGI ≤ 70 or PGI/PGII ≤ 4	68.3	77.1
PGI ≤ 70 or PGI/PGII ≤ 5	73.1	69.2
PGI ≤ 70 or PGI/PGII ≤ 6	81.7	64.2

### The clinical value of serum PG levels in the diagnosis of peptic ulcer

As shown in Table 1, serum PG I level in the patients with gastric ulcer and those with duodenal ulcer were 147.58 ± 57.81 μg/L (n = 36) and 217.43 ± 51.12 μg/L (n = 31), respectively, significantly higher than in the subjects with endoscopically normal mucosa (118.39 ± 47.8 μg/L). These data suggest that serum PG I level is useful in the differential diagnosis of gastric cancer from gastric ulcer, and increased PG I levels is a risk factor for peptic ulcer.

### The association of *H. pylori* infection and serum PG levels

Finally, we detected the infection of *H. pylori* in the subjects. The rate of *H. pylori* infection was 52.9% (18/34) in healthy controls, 67.3% (37/55) in superficial gastritis patients, 80.0% (16/20) in atrophic gastritis patients, 85.4% (70/82) in gastric cancer patients, and 89.6% (60/67) in duodenal ulcer patients. The infection rate was significantly higher in diseased group than in healthy controls (p < 0.01).

In addition, we found that serum PG levels were significantly higher in Hp + group than in Hp- group, while PG I/II ratio was significantly lower in Hp + group than in Hp- group (p < 0.05, Table 5). There data suggest that H. pylori infection is associated with increased serum PG levels.

**Table 5:** **Serum PG I level and PG I/II ratio in ***H. pylori*** positive and negative group**

**Group**	**N (%)**	**PGI (μg/L)**	**PG I/PG II**
Hp +	201 (77.9)	174.82 ± 41.73*	6.74 ± 1.72*
Hp -	57 (22.1)	101.23 ± 10.77	10.08 ± 3.31

*p < 0.05 vs. Hp – group.

## Discussion

In this study, our results showed that serum PG I level and PG I/II ratio decreased in patients with CAG compared with those with NAG, although no statistical significance was observed. However, serum PG I level and PG I/II ratio in patients with advanced gastric cancer and PG I/II ratio in patients with early gastric cancer were lower than those with CAG. These results are in accordance with previous reports [3,4]. Serum PG I concentration decreases with the progression of gastric atrophy as well as gastric cancer because of the loss of chief cells in the fundic glands.

By using the ROC curve, we analyzed the correlation between the diagnostic accuracy based on serum PG levels with gastroscopy and histologic assessment. The area under ROC curve (AUC) is used to measure the ability of each biomarker to differentiate gastric cancer patients and non gastric cancer patients. The results showed that low PG I level and low PG I/II ratio were valuable serologic markers for predicting gastric cancer, especially low PGI/II ratio was effective parameter for screening individuals at high risk of early gastric cancer.

Serum PG levels are known to be affected by demographic factors including sex, age, smoking, drinking, and dietary habits, PG test methodologies such as radioimmunoassay or enzyme immunoassay, which could explain various cut-off values of serum PG profiles in different populations [14]. For instance, in Japan, the proposed cut-off points to determine atrophy and gastric cancer risk are 70 μg/L for PG I and 3.0 for PGI/II ratio [15]. In European countries, the cut-off values were 25 μg/L for PG I and 3.0 for PG I/II [16]. In a Korean study, PG I ≤ 70 ng/mL showed sufficient sensitivity (72.4%) but a low specificity (20.2%), and the sensitivity and specificity of a PGI/II ratio cut off of ≤ 3 were 59.2-61.7% and 61.0%, respectively [17]. In addition, different test-systems for serum PG levels are generally used in different parts of the world. For example, ELISA is used mainly in Europe while latex agglutination test is commonly used in Japan. The use of different test methods could bring potential discrepancy in serum PG levels [18].

In this study, all subjects were Chinese Han people in Hunan who lived in a similar environment, had similar eating habits and life style, and were of the same race. In addition, there were no significant differences in age and sex between patients with different gastric diseases and the controls. Our findings showed that both a low PG I level and PG I/II ratio could indicate the occurrence of gastric cancer. The optimal cut-off value in this population for the diagnosis of gastric cancer was 70 μg/L for PG I with 82.1% sensitivity and 72.5% specificity, and 6.0 for PG I/II ratio with 82.9% sensitivity and 76.8% specificity. The sensitivity and specificity were 62.1% and 94.2%, respectively, when combing PG I level and PG I/II ratio. These results suggest that combined use of low PG I level and PG I/II ratio are valuable for predicting gastric tumors. While the optimal cut-off value of 70 μg/L for PG I was similar to that reported in Japan and Korea, the cut-off value of 6.0 for PG I/II ratio was higher than 3.0 reported in Japan and Korea [15,17]. On the other hand, the cut-off values were 25 μg/L for PG I and 3.0 for PG I/II in European countries [16]. The difference between our data and previous data may be due to the different methodology used to detect PG levels, but also could be due to ethnic background of Chinese.

In addition, in this study we found that gastric ulcer rarely occurred in this population with serum concentration of PG I ≤ 100 μg/L or PG II ≤ 10 μg/L, while duodenal ulcer rarely occurred in those with serum concentration of PGI ≤ 120 μg/L or PG II ≤ 9 μg/L. These results suggest that increased serum PG levels are higher risk of peptic ulcer. It has been reported that hyperpepsinogenaemia may be considered as a subclinical marker of the genetic predisposition to duodenal ulcers [19]. Furthermore, we found that *H. pylori* infection was associated with increased serum PG levels, in agreement with previous studies [19-21]. It is known that infection rate of *H. pylori* is extremely high in gastric duodenal ulcer. Therefore, serum PG test is a useful method for the screening and diagnosis of peptic ulcer.

The limitations of this study should be pointed out. First, the sample size of this study is relatively small. Second, we only examined the population who live in local area of Hunan, midsouth of China. Our results may not represent the whole Chinese population. Therefore, further multi-center studies that employ large-scale subjects are needed to confirm our findings reported in this study.

## Conclusions

Our study suggests that serum PG I level and PG I/II ratio are valuable markers of gastric mucosal changes (as “serologic biopsy”). Serum PG non-invasive tests could provide a tool for selecting the population at high risk of gastric cancer, and reduce the cost and efforts of endoscopy during large scale gastric cancer screening. However, because the sensitivity and specificity of PG test are different in different areas and populations, further studies are necessary to increase the efficacy of PG test for gastric disease diagnosis.

## Competing interest

The authors declare that they have no competing interests.

## Authors’ contribution

XZ carried out most of the experiments and drafted the manuscript. XL collected the samples. HG performed the statistical analysis. JL and GZ conceived the study. All authors read and approved the final manuscript.

## Pre-publication history

The pre-publication history for this paper can be accessed here:

http://www.biomedcentral.com/1471-230X/14/3/prepub
